# Synthetic Microwave Focusing Techniques for Medical Imaging: Fundamentals, Limitations, and Challenges

**DOI:** 10.3390/bios14100498

**Published:** 2024-10-12

**Authors:** Younis M. Abbosh, Kamel Sultan, Lei Guo, Amin Abbosh

**Affiliations:** 1 College of Electronics Engineering, Ninevah University, Mosul 41002, Iraq; younis.abbosh@uoninevah.edu.iq; 2 School of EECS, The University of Queensland, St Lucia, QLD 4072, Australia; l.guo3@uq.edu.au (L.G.); a.abbosh@uq.edu.au (A.A.)

**Keywords:** synthetic focusing, electromagnetic imaging, microwave imaging, medical imaging, delay and sum, confocal imaging, time reversal

## Abstract

Synthetic microwave focusing methods have been widely adopted in qualitative medical imaging to detect and localize anomalies based on their electromagnetic scattering signatures. This paper discusses the principles, challenges, and limitations of synthetic microwave-focusing techniques in medical applications. It is shown that the various focusing techniques, including time reversal, confocal imaging, and delay-and-sum, are all based on the scalar solution of the electromagnetic scattering problem, assuming the imaged object, i.e., the tissue or object, is linear, reciprocal, and time-invariant. They all aim to generate a qualitative image, revealing any strong scatterer within the imaged domain. The differences among these techniques lie only in the assumptions made to derive the solution and create an image of the relevant tissue or object. To get a fast solution using limited computational resources, those methods assume the tissue is homogeneous and non-dispersive, and thus, a simplified far-field Green’s function is used. Some focusing methods compensate for dispersive effects and attenuation in lossy tissues. Other approaches replace the simplified Green’s function with more representative functions. While these focusing techniques offer benefits like speed and low computational requirements, they face significant ongoing challenges in real-life applications due to their oversimplified linear solutions to the complex problem of non-linear medical microwave imaging. This paper discusses these challenges and potential solutions.

## 1. Introduction

Medical microwave imaging involves illuminating tissues with signals at microwave frequencies and collecting the reflected/scattered waves. Synthetic focusing techniques enhance spatial resolution by synthesizing an antenna array from a single transmitting/receiving element or using an antenna array where each antenna element operates as a transmitter sequentially [1,2,3]. By successively exploring signals collected at different antenna positions or by different antenna elements of the array, an image of the target’s scattering properties is constructed.

Researchers working on microwave imaging have used many focusing techniques, such as confocal imaging, time reversal, back-projection, and delay-and-sum methods [4,5,6,7,8,9,10]. All of these methods can be classified as synthetic focusing techniques, which are qualitative methods [11]. They are based on beamforming methods using the concept of synthetic aperture radar. Some of these methods can be linked to the special filtering concept [12]. Essentially, the imaged object is illuminated by a single antenna that is moved successively to different positions or an array of antennas that are excited individually and sequentially. For the single-antenna approach, the antenna at each position transmits a microwave signal and receives the reflected signal, which is converted into digital form and stored. The measured signals are summed in the processing unit after the proper time (or phase) shifting. If the shifting is correctly performed, only the scattered signals arriving from the strong scatterer within the imaged domain contribute constructively to the sum. On the contrary, the scattered signals due to other areas of the imaged domain add destructively, and their contributions appear with very low intensity and thus appear as noise in the final image.

Focusing techniques are qualitative, identifying the presence and location of targets by their scattering signature [13,14,15]. They do not aim to generate a quantitative, detailed image of the domain, such as tomography [8,16,17]. By properly combining the receiving signals at one antenna at different locations or using an array of antennas transmitting sequentially, a qualitative image of the domain can be created, pixel by pixel, revealing any strong scatterer within that domain. This can be done simply by changing the time-shifting values added to the signals received at the various measurement positions of the scanning antennas. The main difficulty inherent in this simple approach is that, for focusing on a specific space point within the imaged domain, the various shifting coefficients must be defined with knowledge of the propagation velocity inside the propagation medium [18]. However, the wave velocity inside the imaged object is usually unknown, as the exact dielectric properties of that domain are not known. Thus, some a priori information about the imaged object is used to give a reasonable estimate of the wave velocity in the imaged object [19]. Using one average velocity means the imaged domain is assumed to be homogeneous, while in medical imaging, it is not.

Many researchers invested significant efforts to improve the basic formulation of focusing microwave techniques on medical applications. In some cases, the known information about the imaged object, such as the dielectric properties of human tissues, is utilized [20]. In others, the reflections due to the external boundaries of the scatterer under test are either removed or compensated [21]. For example, assuming the external shape of the object and the antenna–object distance is known, the scattered or reflected signals from the object’s external boundaries can be deduced and eliminated. In other cases, however, this distance is not known. For example, in medical imaging, where there is the need to compensate for the reflection from the air–skin interface, the body’s shape is patient-dependent. Consequently, other procedures were developed to deduce the first reflection from the target [22]. Another important issue concerns the compensation of dispersion effects in signal propagation, which can introduce notable broadening in the pulse duration [22,23,24,25,26]. In medical applications, an approach based on a broadband beamformer implementing frequency-dependent amplitudes and phase changes in the various channels was proposed to address the dispersive behavior of tissues [5].

The focusing techniques can be applied in time or frequency domains [22,23,24]. In the time domain, an ultrawideband transmitter emits the microwave signal, which scatters within the propagation medium. Multiple receiving antennas collect the scattered waves. By combining received signals appropriately, the response from a limited zone inside the scatterer is obtained, allowing pixel-by-pixel exploration of the entire target. A signal that sweeps across a specific frequency band is transmitted in the frequency domain. Reflected and/or scattered signals at each frequency are collected. These are then processed separately in the final domain before summing up the results. Alternatively, they can be transformed into the time domain and processed.

**Figure 1:**
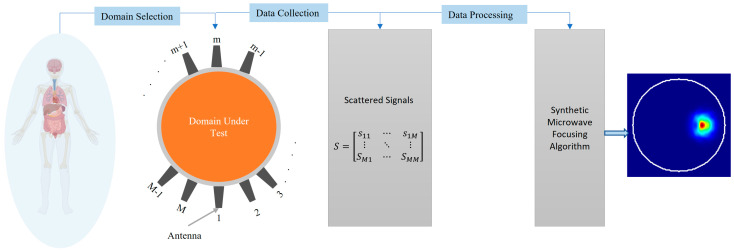
Concept of medical imaging using synthetic microwave focusing techniques.

This paper explores the fundamental principles, challenges, and advancements in synthetic microwave-focusing techniques for medical imaging (Figure 1). It provides a critical overview that can guide future research and development. The insights offered can help address the technical hurdles that currently limit the wide practical application of these techniques, such as the need for more accurate models and the complex nature of electromagnetic wave propagation in biological tissues. Ultimately, this paper aims to potentially transform microwave imaging into a mainstream diagnostic tool that can improve patient outcomes and reduce healthcare costs.

The paper is organized as follows: Section 2 explains that the scalar electromagnetic scattering theory provides the foundational principles for understanding synthetic microwave focusing techniques. The transition from scalar electromagnetic scattering theory to focusing techniques, summarizing how these theoretical concepts are applied in practical imaging methods, are explained in Section 3. Section 4 explores the different focusing techniques, including confocal imaging, time reversal, back-projection, and delay-and-sum methods, detailing their unique approaches and applications. Section 5 focuses on the challenges and solutions associated with focusing techniques for medical imaging, addressing issues such as tissue heterogeneity, multi-scattering effects, and the need for knowledge of precise propagation velocity. Section 6 evaluates the performance of the focusing techniques within the same domain under test. Section 7 provides a summary of the paper.

## 2. Scalar Electromagnetic Scattering Theory

The scalar EM wave equation for propagation in a two-dimensional medium that has Green’s function ($G(r,r^{\prime })$) is [26]:

(1) $$\nabla^{2}E_{t}+k^{2}E_{t}=0$$

(2) $$\left(\nabla^{2}+k^{2}\right)G\left(r,r^{\prime }\right)=-\delta \left(r-r^{\prime }\right)$$

where $E_{t}$ is the total electric field, k is the wavenumber in the medium, and $\delta \left(\cdot \right)$ is an impulse function.

If the medium includes a scatterer of area (A), (1) and (2) can be solved to find the scattered field as:

(3) $$E_{s}\left(r\right)=\iint_{A}G\left(r,r^{\prime }\right)\chi \left(r^{\prime }\right)E_{t}\left(r^{\prime }\right)dr^{\prime }$$

where χ(·) is the dielectric contrast between the scatterer and the background medium. Using Born approximation [26]:

(4) $$E_{s}\left(r\right)\approx \iint_{A}G\left(r,r^{\prime }\right)\chi \left(r^{\prime }\right)E_{i}\left(r^{\prime }\right)dr^{\prime }$$

where $E_{i}$ is the incident electric field.

If the scatterer is small compared to the wavelength, the above analysis can be approximated as follows. Let $J(r_{o})$ be the current density at the locations of the transmitting dipoles ($r_{o}$). Assume the propagation medium has Green’s function $G(r_{o},r_{s})$. The incident field at the location of the scatterer $\left(r_{s}\right)$ is:

(5) $$E_{i}\left(r_{s}\right)=\iint_{S}J\left(r_{o}\right)G\left(r_{s},r_{o}\right)dr_{o}$$

where S is the area that contains the dipole. When only one dipole is considered, the incident field can be further simplified as:

(6) $$E_{i}\left(r_{s}\right)=J\left(r_{o}\right)G\left(r_{s},r_{o}\right)$$

The current density at $r_{o}$ is related to the dipole moment $p(r_{o})$ as:

(7) $$J\left(r_{o}\right)=j\omega p\left(r_{o}\right)$$

In addition, for a scatterer with permittivity ($\epsilon_{s}$) in a background medium with permittivity ($\epsilon_{b}$), the dipole moment is:

(8) $$p\left(r_{s}\right)=\epsilon_{b}\alpha E_{i}\left(r_{s}\right)$$

where the polarizability (α) for small scatterers is [23]:

(9) $$\alpha =\frac{3V}{4\pi }\frac{\Delta \epsilon }{\epsilon_{b}+2\epsilon_{s}}$$

Δϵ is the difference between the dielectric constant of the scatterer and the background medium. The scattered field $E_{s}(r)$ at a point (r) is given by:

(10) $$E_{s}\left(r\right)=\mu_{0}\omega^{2}G\left(r,r_{s}\right)p\left(r_{s}\right)$$

Substituting the expressions for $p(r_{s})$ and $E_{i}(r_{s})$, the scattered field can be expressed as:

(11) $$E_{s}\left(r\right)={j\mu }_{0}\omega^{3}\epsilon_{b}\left(\frac{3V}{4\pi }\frac{\Delta \epsilon }{\epsilon_{b}+2\epsilon_{s}}\right)G\left(r,r_{s}\right)G\left(r_{s},r_{o}\right)p\left(r_{o}\right)$$

Assuming a homogeneous medium [19,20]:

(12) $$G\left(r,r_{s}\right)=\frac{e^{ik|r-r_{s}|}}{|r-r_{s}|}$$

(13) $$G\left(r_{s},r_{o}\right)=\frac{e^{ik|r_{s}-r_{o}|}}{|r_{s}-r_{o}|}$$

where (k) is the wave number of the medium.

Equation (11) shows the scattered field $E_{s}(r)$ at a point (r) as a function of the dipole moment $p(r_{o})$, the Green’s functions of the medium, and the properties of the scatterer and the medium. If the medium includes n scatterers, the total scattered field at r, while ignoring any multi-scattering or interaction between those objects, will then be:

(14) $$E_{s}\left(r\right)={j\mu }_{0}\omega^{3}\epsilon_{b}p\left(r_{o}\right)\sum_{i=1}^{n}N_{i}G\left(r,r_{si}\right)G\left(r_{si},r_{0}\right)$$

where:

(15) $$N_{i}=\left(\frac{3V_{i}}{4\pi }\frac{{\Delta \epsilon }_{i}}{\epsilon_{b}+2\epsilon_{si}}\right)$$

According to (14), the phase shift from an antenna position to a certain imaging point depends on the Green’s function from the antenna to the scatterer and the Green’s function from the scatterer to the imaging point. Both of those factors depend on the complex permittivity of the medium.

## 3. From Scalar EM Scattering Theory to Focusing Techniques

In the focusing techniques, the received signals are usually amplitude-compensated and phase-corrected to account for the signal attenuation and travel time differences. If there are M measurement points or M antennas, the focused intensity image at any location, r, within the imaged domain I(r) is obtained by coherently summing the amplitude-compensated and phase-corrected scattered signals:

(16) $$I\left(r\right)=\sum_{m=1}^{M}{A\left(r,r_{m},\epsilon_{b}\left(f\right),\epsilon_{s}\left(f\right)\right)E}_{s}\left(r_{m}\right)e^{ik\tau \left(r,r_{m},\epsilon_{b}\left(f\right),\epsilon_{s}\left(f\right)\right)}$$

where $A\left(r,r_{m},\epsilon_{b},\epsilon_{s}\right)$ accounts for the compensation to signal attenuations and $\tau (r,r_{m},\epsilon_{b},\epsilon_{s})$ accounts for the phase delay or travel time. Both $A\left(\cdot \right)$ and τ(·) are functions of $\epsilon_{b}\left(f\right),$ which is usually unknown in an imaging task; thus, the analytical calculation of $A\left(\cdot \right)$ and τ(·) is a challenging task.

In most focusing techniques, such as confocal and DAS methods [24,27], $\epsilon_{s}$ and $\epsilon_{b}$ are assumed to be an estimated averaged value, and the frequency dependency (i.e., dispersive feature) is neglected. Consequently, the phase delay τ(·) can be calculated using the round-trip time from the antenna to the focusing point. In other methods, such as [28], different antennas are assigned with different values of $\epsilon_{b}$. Hence, Equation (16) is modified to:

(17) $$I\left(r\right)=\sum_{m=1}^{M}\;{A\left(r,r_{m},\epsilon_{b}\left(m\right),\epsilon_{s}\right)E}_{s}\left(r_{m}\right)e^{ik\tau \left(r,r_{m},\epsilon_{b}\left(m\right),\epsilon_{s}\right)}$$

Note that the dispersive feature of $\epsilon_{b}$ and $\epsilon_{s}$ is neglected in (17). The value of $\epsilon_{b}\left(m\right)$ can be obtained through an optimization-based approach, as explained in [28].

According to (14)–(16), the signal attenuation from the antenna position to a certain imaging point stems from the two Green’s functions, the Green’s function from the antenna to the scatterer and Green’s function from the scatter to the imaging point. In other words, the signal attenuation depends on three factors: the distance between the antenna and the scatterer, the distance between the scatterer and the imaging point, and the complex permittivity of the background medium ($\epsilon_{b}$). Therefore, to accurately compensate for the signal attenuation, the dielectric properties of the background medium are needed.

Determining the compensation function $A\left(\cdot \right)$ requires an assumed signal model. For example, a path loss factor is calculated using the square of the distance between r and $r_{m}$ [29]. This method correctly considered the signal attenuation caused by the two Green’s functions; however, the attenuation caused by the complex permittivity of the background medium is not included. In [30], a far-field approximation was used to build a frequency-dependent attenuation factor in an exponential function to account for the attenuation caused by the background medium. However, only one order of the distance between r and $r_{m}$ was used in that model, which implies that only one Green’s function was used to account for the signal attenuation. In [31], the continuous wavelet transform was used to build $A\left(\cdot \right)$ in a time-reversal method.

To address those challenges, a hybrid method, which combines the focusing techniques with a quantitative (tomography) algorithm, was proposed to achieve a better a priori knowledge of $\epsilon_{b}(f)$ [32]. In the hybrid method, a general distribution of $\epsilon_{b}$ is calculated from a quantitative algorithm, and then a better phase correction and compensation for signal attenuation can be achieved by using the calculated $\epsilon_{b}$ map.

## 4. Types of Focusing Techniques

There are different ways to implement (16) in medical microwave imaging. However, all of those methods use the same concept but with different assumptions.

**Figure 2:**
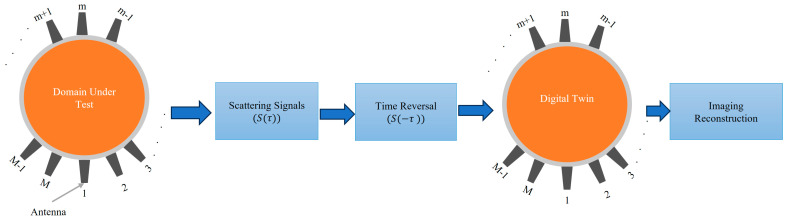
Block diagram of the time reversal microwave imaging concept.

Time Reversal (TR): This technique involves irradiating the imaged domain, recording the scattered waves, reversing them in time, and re-transmitting them to focus on the source of scattering within the domain (see Figure 2) [33,34,35]. The TR method leverages the principle that the imaging domain is reciprocal and that the relevant EM problem has two solutions: E(t) and E(−t). Thus, the electromagnetic waves can be reversed in time to refocus on their source. A microwave signal is transmitted from an antenna and travels through the imaged domain, interacting with various scatterers within that domain. The scattered waves are then recorded by an antenna array placed around the imaged domain. Those recorded signals are then time-reversed and re-transmitted into a numerical model that emulates the experiment. The intensity of the signal will be maximum at the location of the scatterer or strongest scatterer and can be calculated as follows [33,34].

(18) $$I_{TR}\left(r\right)=\sum_{i=1}^{M}\left|\int_{0}^{T}\;S_{i}\left(t\right)\cdot G\left(r,r_{i},T-t\right)dt\right|$$

where M refers to the total number of scattered signals. S(t) refers to the scattered signal, and the integration represents the time reversal process, while $G\left(r,r_{i},t\right)$ refers to the Green’s function representing the wave propagation from the antenna at $r_{i}$ to the imaging point at r.

Most of the literature uses a simplified version of the TR to avoid the complexity of the calculated green function and the difficulty of the measurement, assuming that the integration of the green function can be simplified into $S_{i}^{*}\left(t+\tau_{i}\left(r\right)\right),$ which is reasonably accurate only for homogenous medium or weakly scattering medium.

(19) $$I_{TR}\left(r\right)\approx \sum_{i=1}^{M}S_{i}^{*}\left(t+\tau_{i}\left(r\right)\right)$$

where $S_{i}^{*}(t)$ refers to the time-reversed scattered signal while $\tau_{i}\left(r\right)$ refers to the round-trip time delay from i-th antenna to the point r in the imaging domain. The expression $t+\tau_{i}\left(r\right)$ represents the time-shifting of the signal to refocus on the imaging point.

**Figure 3:**
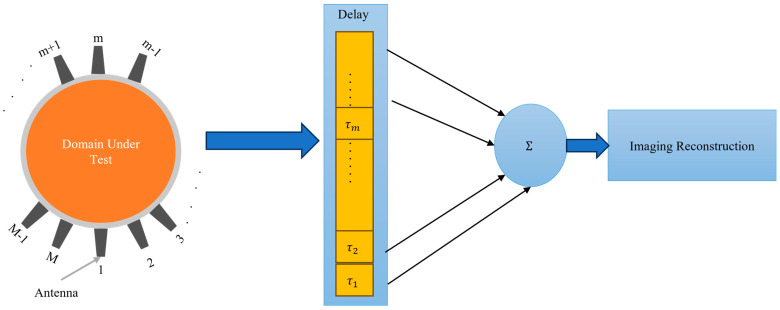
Block diagram of the confocal imaging and Delay-and-Sum concept.

Confocal Imaging: In this approach, the imaged domain is irradiated with a wideband microwave signal from an antenna array surrounding that domain. The scattered signals are collected by those antennas and processed to synthetically focus on different points within the domain [36,37]. This involves time-shifting and summing the signals to enhance the reflections from specific locations (see Figure 3). By coherently adding the scattered signals from strongly scattering objects, such as tumors, an image is formed to highlight areas with significant dielectric contrast, such as between healthy and cancerous tissue.

(20) $$I_{confocal}\left(r\right)=\sum_{i=1}^{M}{\left.|S_{i}\left(t-\tau_{i}\left(r\right)\right)\right|}^{2}$$

Delay-and-sum: This technique, which is effectively the same as the confocal imaging algorithm shown in Figure 3, involves delaying the received signals by appropriate time and summing them to enhance the signal from the target [38]. Simple DAS techniques cannot compensate for frequency-dependent propagation effects, such as dispersion. They also suffer from the adverse effects of artifacts and noise. In this technique, multiple antennas transmit wideband microwave pulses into the imaged domain. The scattered signals from the domain are received by those antennas. The received signals are time-shifted (delayed) to account for the travel time from the antennas to each point in the imaging domain. The time-shifted signals are summed together as expressed in (21). This process enhances the signals from the true scatterers while reducing noise and interference. The summed signals are used to construct an image, highlighting areas with significant dielectric contrast, such as between healthy and malignant tissues. The DAS method is valued for its simplicity. The only difference between confocal imaging and DAS is the squaring operation in confocal imaging, which enhances the intensity of the scattering signals, providing higher contrast for strong scatterers.

(21) $$I_{DAS}\left(r\right)=\sum_{i=1}^{M}\;S_{i}\left(t-\tau_{i}\left(r\right)\right)$$

**Figure 4:**
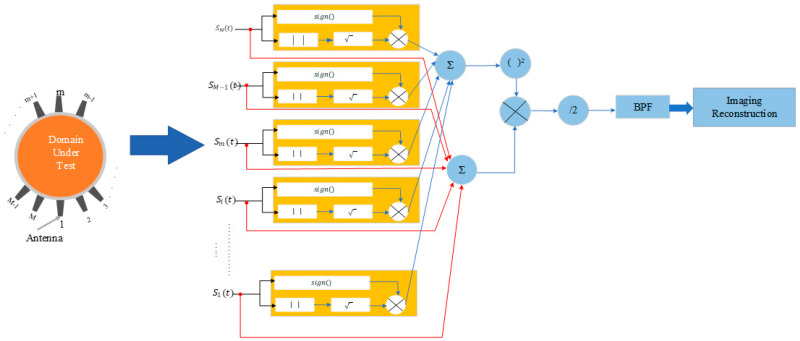
Block diagram of the Delay-Multiply-and-Sum algorithm.

There are several variations to the simple DAS algorithm. For example, the Delay Multiply and Sum (DMAS) [7,27] builds on DAS by introducing a multiplication step before summation, which helps to improve the imaging of weak scatterers by enhancing the non-linear response of the algorithm (see Figure 4). This approach increases the contrast and reduces the impact of noise and clutter. The imaging intensity of the DMAS is expressed as

(22) $$I_{DMAS}\left(r\right)=\sum_{i=1}^{M-1}\;\sum_{j=i+1}^{M}\;S_{i}\left(t-\tau_{i}\left(r\right)\right)\cdot S_{j}\left(t-\tau_{j}\left(r\right)\right)$$

Many studies have tried to address the limitations of traditional DAS methods [14,27,39,40,41,42,43]. Key improvements include the Improved Delay-and-Sum (IDAS) technique, which uses a weighting factor for each channel at each focal point to enhance target signals and reduce noise. The Coherence Factor Delay-and-Sum (CF-DAS), adapted from ultrasound imaging, uses a normalized factor based on backscattered signals to improve image quality. The Channel Ranked Delay-and-Sum (CR-DMAS) scales each channel by a weighting factor based on the propagation path length. These modifications are summarized in [44]. Techniques to remove artifacts, such as rotation, averaging, adaptive filtering, differential, and neighbor-based methods, are adopted [45,46]. Further advancements include Fast DMAS, which optimizes computational efficiency for real-time applications [13,14,18], and multi-static double-stage DMAS, which uses multiple static antennas to enhance spatial resolution and accuracy [7].

Back projection: Since the forward projection operation mapped scatterers inside the domain into the scattered signals, the back projection operation maps those signals back to the scatterers inside the domain. So, back-projection techniques reconstruct the image by projecting the scattered signals back into the imaging domain [47,48,49].

## 5. Performance Evaluation of Synthetic Microwave Focusing Algorithms

**Figure 5:**
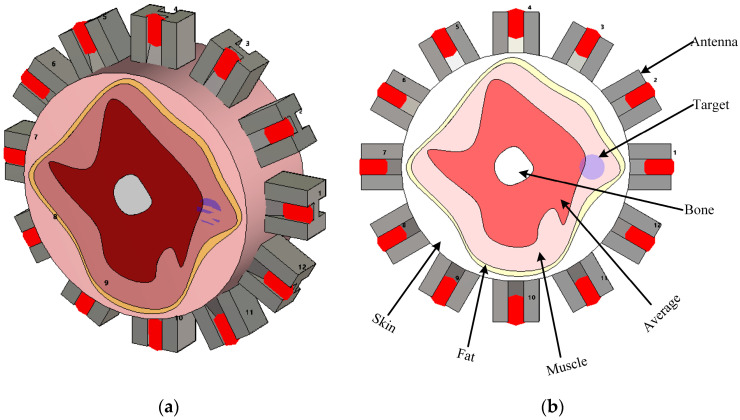
Simulation environment representing a general human organ to assess different focusing techniques. (**a**) 3D view and (**b**) detailed view.

To compare and assess the effectiveness of the abovementioned synthetic microwave focusing techniques, an imaging domain, as shown in Figure 5 to represent a general medical imaging scenario, is built in CST Microwave Studio. A setup consisting of 12 dielectric-loaded antennas, carefully matched to the domain under test, is employed [50]. These antennas are positioned to encircle the domain, ensuring full coverage and improving imaging resolution (see Figure 5). Operating over a broad bandwidth of 0.7 GHz to 2 GHz, the antennas were capable of capturing detailed electromagnetic interactions across the frequency range, which is critical for distinguishing different tissue types. The domain includes the most common tissues found in human organs, such as skin, fat, muscle, and bone, while the remaining tissue within the domain is modeled using average dielectric properties representative of typical human tissues. The dielectric properties of those tissues over the interested frequency range are shown in Table 1 [51]. The domain under test has a radius of 80 mm, providing a realistic scenario for imaging human anatomical structures. The same setup is used to simulate an abnormality within the domain but with the inclusion of a target representing a 7.5 mm radius anomaly. The dielectric properties of the target are chosen to be water, emulating a physiological abnormality, such as a tumor or lesion, within the imaged object. This approach allows the use of a controlled environment to study the focusing techniques’ ability to accurately detect and localize anomalies based on their electromagnetic scattering properties.

The focusing techniques explained above are used to generate images using the simulation in Figure 5. All techniques follow the scalar solution to the problem as represented by (16) and assume the effective permittivity, $\varepsilon_{b}$, which is set to 42 after the parametric study. This value, representing the average effective permittivity for the domain, gives optimal possible imaging quality. The investigated techniques were run using MATLAB R2023a in a Dell Intel Xeon W-2123 CPU workstation with 32 GB RAM. The computation time for confocal, DAS, DMAS, and time-reversal methods were 1, 10, 15, and 30 s, respectively, which confirm the fast processing using focusing techniques on a general computational platform. The TR method has the longest processing time compared to other focusing techniques due to the need to calculate the inverse propagation for each antenna.

**Figure 6:**
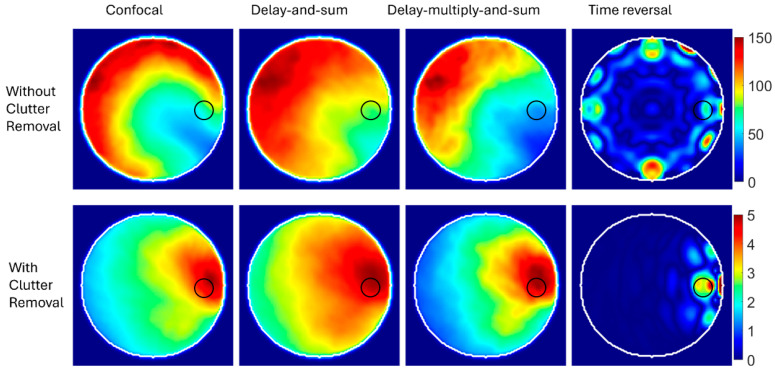
Reconstructed images using synthetic focusing techniques with and without clutter removal.

The imaging results are shown in Figure 6. For data processing without using a clutter removal method, all the focusing techniques fail to detect and localize the abnormality. The strong assumptions used in those techniques make them incapable of generating reasonable images from scattered signals generated from a heterogeneous medium. So, in this case, the simplicity and limited computational resources required by those algorithms do not mean much when it comes to medical applications where accuracy is critical.

The results are different if a priori information about the domain is used before applying those techniques. For example, a differential algorithm is used to remove clutter by subtracting the scattered signals in a healthy copy of the imaged domain from the unhealthy copy. The synthetic focusing techniques are then used to process clutter-free signals. The results, shown in the lower row of Figure 6, indicate the capability of those techniques to detect and localize abnormalities. The color scale of the generated images after clutter removal indicates a lower intensity than images generated without removing clutter. This is because clutter is the strongest signal, and thus, signals after removing clutter have a much lower intensity.

To further assess the diagnostic accuracy of the imaging results shown in Figure 6, the positioning error, as defined in [28], is calculated. Since all the focusing techniques failed to detect the target when no clutter removal techniques were used, only their reasonable imaging results after removing clutter were assessed. The error in the center of the detected target compared to the ground truth was 12.8, 14.6, 14.6, and 7.6 mm for confocal, DAS, DMAS, and TR, respectively. While the results indicate that TR gives the best results, we must remember that TR requires longer processing time and a perfect digital twin for the imaged domain, which is usually unavailable in real-life scenarios. The DAS and DMAS have identical positioning errors. This result agrees with the theoretical definitions of DAS and DMAS, which indicate that DMAS cannot improve target localization but can, to some degree, enhance the signal-to-clutter ratio.

The above results confirm that while synthetic focusing techniques are fast and require limited computational resources, they are derived for a homogeneous medium. They are adopted from the theory of free-space synthetic aperture radars used for remote sensing. Thus, they are not directly applicable to the complex mission of imaging heterogeneous tissues. One way to make them workable in medical imaging is by using thorough a priori information about the imaged object, such as a “healthy” copy of the imaged object. However, this type of information is generally not available. Thus, other solutions should be investigated to enable using those techniques in real-life medical imaging.

## 6. Focusing Techniques for Medical Imaging: Challenges and Solutions

Microwave-focusing imaging techniques offer several advantages, including simplicity and speed. These methods are efficient and can quickly produce images without extensive computational resources. However, they also encounter significant challenges that impact their accuracy and reliability in practical scenarios. These challenges include the need for precise knowledge of the propagation velocity within the imaged object, the heterogeneous nature of biological tissues, and the assumptions of small, independent scatterers with low dielectric contrast. Additionally, issues such as reflections from external boundaries, multi-scattering within the object, and frequency dispersion of tissues further complicate the imaging process. Addressing these challenges is crucial for improving the effectiveness of microwave-focusing imaging techniques in real-life applications.

a. Unknown propagation velocity

To focus on specific spatial points, time-shifting coefficients must be defined based on propagation velocity. However, the accurate wave velocity inside the imaged object is unknown as the dielectric properties of that object are not accurately known. One possible solution to address this challenge is the use of information about the dielectric properties of the domain for the rough estimation of the wave velocity [52,53,54]. Moreover, calibration phantoms with known dielectric properties [55] can be used to calibrate the imaging system and improve the accuracy of velocity estimation.

b. Heterogeneous medium

Biological tissues are heterogeneous, causing the propagation environment for electromagnetic waves to be complex, while focusing techniques often assume a homogeneous medium. To address this challenge, the focusing techniques can be revised to make them adaptive in a way that can account for heterogeneity. For example, time-reversal imaging can adaptively focus on scatterers by iteratively refining the model based on the received signals [56]. Another potential solution is to use multifrequency approaches to capture the varying dielectric properties across different frequencies, providing a more accurate representation of the heterogeneous medium [57].

c. Dependent scattering

The derivation of focusing techniques assumes small, independent scatterers with low dielectric contrast (Born approximation) in the imaging domain. To address this challenge in some scenarios, more advanced scattering models might be employed. For example, the Rytov approximation [58] or non-linear inverse scattering methods [16,59,60,61] can handle higher dielectric contrasts and multiple scattering effects. Also, the combined Born–Rytov method could improve imaging compared to using either approximation separately [62]. However, adopting those approaches might remove the advantages of the focusing techniques concerning simplicity and speed. Another potential solution is using the digital twin. In this case, a realistic full-wave simulation environment is built to model the complex interactions within the medium more accurately.

d. Reflections from external boundaries

Compensating for reflections from external boundaries is crucial to avoid artifacts in the reconstructed image. External boundaries of the scatterer introduce reflections that dominate the target response. In medical imaging, patient-dependent body shapes require specific procedures for reflection compensation. One way to address this is to implement an accurate boundary estimation method [63], which enables estimating and removing those reflections using proper clutter-removal techniques. Also, time-gating techniques can be used to isolate the primary scattered signals from reflections, improving the clarity of the reconstructed image [64].

e. Multi-scattering compensation

The imaged domain might include several scatterers, causing multi-scattering within the object and complicating the imaging process. To address this challenge, iterative reconstruction algorithms that can account for multiple scattering effects might be used [40]. These algorithms aim at iteratively refining the image by comparing the measured and simulated scattered fields. However, that would require an accurate simulation model. Another way is to develop an AI technique that learns complex scattering patterns and improves the accuracy of the reconstructed images [65,66].

f. Frequency dispersive properties

Biological tissues are frequency dispersive, meaning their permittivity varies with frequency. Thus, the wide frequency band used in the focusing technique would have different wave velocities and, thus, different time delays, knowing that dispersion broadens pulse duration during signal propagation. One way to address this challenge is to use the Debye or Cole–Cole model as part of the time delay estimation [67]. These models account for the dispersive nature of tissues and can be incorporated into imaging algorithms. Also, broadband beamformers with frequency-dependent amplitudes and phase adjustments could mitigate dispersion effects.

g. Simplified Green’s function

While most algorithms use the simplified Green’s function for a homogeneous domain, it is not accurate for medical imaging of heterogeneous tissues. A possible solution is to use a more representative function of the imaged domain, such as the Bessel function in head imaging [23] or the Henkel function in torso imaging [67]. Another solution that is not easy to implement is using numerical methods to compute Green’s functions for heterogeneous media. Techniques like the finite element method or finite difference time domain can be used to calculate Green’s functions that account for the complex structure of biological tissues. Also, Green’s function can be experimentally calibrated using known reference objects to improve its accuracy for specific imaging scenarios. The side effect of this approach is a significant increase in the processing time and required computational resources, which might mask the benefits of synthetic focusing techniques.

**Figure 7:**
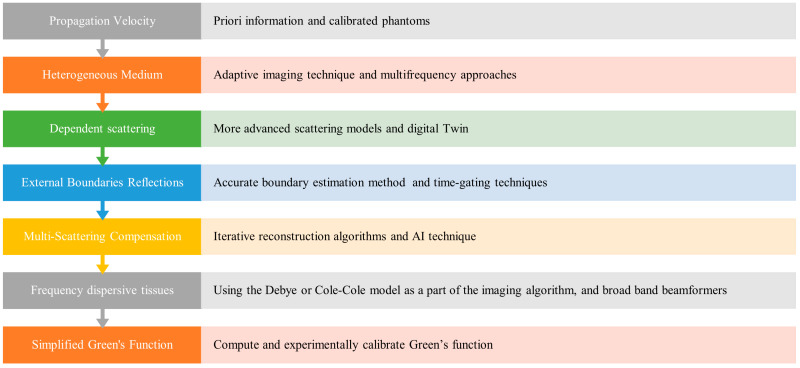
Challenges and Solutions in Synthetic Microwave Focusing Techniques for Medical Imaging.

Figure 7 summarizes the key challenges encountered in synthetic microwave focusing techniques and their corresponding solutions, highlighting the suggested approaches to enhance the accuracy and effectiveness of medical imaging.

In addition to the solutions mentioned above, synthetic microwave-focusing techniques can be augmented by or combined with other methods to address some of its limitations. In that regard, there are two main areas for further exploration and development as research directions:

i. Integration with Quantitively Imaging Methods

One promising approach involves combining qualitative focusing techniques with quantitative imaging methods [32,64,68,69]. Qualitative techniques can be used to generate preliminary images that can then guide more detailed quantitative analysis. This approach takes advantage of the strengths of both methods, combining the speed and simplicity of qualitative techniques with detailed quantitative methods. Integrating these techniques can improve the overall imaging process, leading to better diagnostic outcomes. For example, initial qualitative images can help identify regions of interest, which can then be examined in greater detail using quantitative methods, thereby improving both efficiency and accuracy.

ii. Augmentation with Deep Learning

Another direction involves combining synthetic focusing techniques with deep learning methods. Deep learning can potentially improve imaging reliability by identifying complex patterns and features from large datasets. This integration can help address the limitations of traditional methods, offering more dependable and accurate imaging solutions, particularly in situations where quick processing is essential. Detection and localization accuracy can be enhanced by training deep-learning algorithms to identify specific patterns related to various types of tissues or anomalies [70,71]. The challenge would be providing the large datasets required for training the deep learning methods.

## 7. Conclusions and Prospects

Synthetic-focusing techniques are qualitative imaging techniques that have been investigated for medical microwave imaging for anomaly detection and localization. While the literature includes many focusing imaging techniques, they are all governed by the same principles of scalar electromagnetic scattering theory. Different assumptions are used in these methods, but they all assume the imaged domain to be homogeneous and non-dispersive. Understanding those assumptions would enable researchers and scientists to understand the limitations of those techniques and potentially use a suitable approach based on their application and main objective. For real-life imaging scenarios, currently used focusing techniques might find it challenging to generate reliable results without having enough a priori information about the imaged domain. Thus, addressing the limitations of those techniques in imaging heterogeneous tissues could improve imaging quality and diagnostic accuracy. Potential methods to address those limitations include thorough calibration, adaptive multi-frequency, digital twin, time gating, frequency dispersive modeling, and representative calibrated Green’s function. Keeping the benefits of focusing techniques concerning simplicity and speed while trying to make them reliable for real-life applications will continue to be a major challenge because of the complex electromagnetic-tissue interaction. One potential direction when processing time is not critical is to use the qualitative focusing imaging technique as an initial image for quantitative imaging methods. Alternatively, augmenting the focusing techniques with well-trained deep-learning methods is an option when speed is crucial.

## Figures and Tables

**Table 1:** Dielectric properties of the tissues included in the tested domain at the frequency band (0.7–2 GHz).

Tissue	Skin	Fat	Muscle	Bone	Human Average	Target
$\epsilon_{r}$ @ (0.7–2 GHz)	42–38.6	11.37–11	55.3–53.3	12.6–117	41.3–36.78	84.5–83
σ (s/m) @ (0.7–2 GHz)	0.8–1.127	0.096–0.21	0.88–1.45	0.12–0.31	0.1–0.65	0.15–0.91

## Data Availability

All the data have been included in the article.
